# The Teeth

**Published:** 1877-04

**Authors:** 


**THE TEETH.**—Natural teeth, clean, sound and perfect, are essential to the comeliness of any human face. Defective teeth mar the handsomest features, and cause us to turn away our gaze with a kind of disgust from a countenance otherwise faultlessly beautiful. Sound teeth not only add to the comfort and personel appearance, but contribute largely to the health of all, hence special and scrupulous attention should be paid to them daily, from early childhood, from the time when the first permanent tooth makes its appearance, about the sixth year.

Whenever it is practicable, every tooth in a child's head should be minutely examined by a careful, conscientious, and skillful dentist every few months; and the great importance of special attention to their cleanliness, the avoidance of cold and hot drinks—of the use of any "picks" harder than wood or quills, and of all dentifrices prepared by unknown hands, should be impressed upon the minds of the young with great assiduity.

Harm has been done by propagating the notion that sugar is injurious to the teeth, by diverting attention from real causes of destruction or decay. The eating of any amount of pure sugar can not injure the teeth directly, because it has no residue, it is wholly dissolved and passes into the stomach.

But let it be remembered that the practice of eating sugars or candies or any other sweetmeats largely, will inevitably cause a disorder of the stomach and generate gases there, which will speedily undermine the health of the teeth.

By insisting too much on the fact that sugars and candies destroy the teeth, an impression will grow that if these are mainly avoided, the person so doing will have good teeth, and this leads the mind away from the necessity of keeping the mouth clean and the stomach healthful. If these things are well done, and the teeth are kept plugged in a finished style, teeth naturally or hereditarily "poor," may be kept in a good state of preservation for many years.

All forms of dyspepsia have a direct tendency to destroy the teeth. Whatever causes acidity of the stomach, is ruinous to the teeth. A tablespoon of the purest syrup of loaf-sugar, taken three times a day before meals, will destroy the tone of the healthiest stomach in a very short time. And when it is remembered how many patent medicines are made up in the form of syrups and sweet lozenges, and how common the use of them has become, it need not be wondered at that every second or third person met on the street knows the meaning of "sour stomach" or dyspepsia. It has been shown that if a sound tooth be steeped in syrup for some days, it becomes a soft, pulpy mass. That does not prove that syrup is injurious to the teeth, because it was a dead tooth; and further, such a steeping of a live tooth is impossible. The gastric juice is innocuous to a living stomach, but at the very moment of death, that same gastric juice begins to eat up the stomach. So it is inconclusive to reason from the living to the dead, or *vice versa*.

It is urged by many that calomel is a most deadly agent to the teeth, and yet if a sound tooth is soaked for weeks together in a solution of calomel, no apparent effect whatever is produced on it.

So far from sugars and pure candies injuring the teeth or the health, they would, if used wisely and in moderation, as sole desserts, be actual preventives of both; especially if alternated, as desserts, with fruits and berries in their natural, raw, ripe, fresh, perfect state, by banishing from our tables the pestiferous pie, the leaden pudding, and pastries and cakes of every name, which, as desserts, always tempt to excesses which lay the foundation for diseases which torture for a lifetime, or bring speedily to the grave.

Let the spirit of this article be distinctly understood. Pure sugars and candies do not injure the teeth, except indirectly, by their injudicious use in exciting acidity of stomach or dyspepsia, as will any other kind of food, or drink, or beverage, if extravagantly used.

At seasons of the year when fruits and berries may not be had, ripe, fresh, and perfect, as desserts, pure sugars and candies may be used as such in their stead to great advantage, because they are healthful, being warming, nutritious, and agreeable; hence, as a table article, they are very valuable, while the almost universal love of them shows that they were intended to be eaten. If a child is not allowed to eat any thing containing sugar it will sicken and die in a very short time. Children need the carbon, the fuel contained in sugar to keep them warm; without it they would perish from cold; hence the love of sweet things is an instinct, implanted by the kind and wise Maker of us all for the child's preservation. There are a parcel of stupid creatures in the world whose sole stock in trade of brains and logic amounts to this, that "whatever is good is unhealthy." It is not advised that children should be allowed to eat sugar and candy whenever they want it; but that as a dessert, after each regular meal, the use of pure sugars and candies would benefit and not injure.

